# Longitudinal patterns of unmet need for contraception among women living with HIV on antiretroviral therapy in South Africa

**DOI:** 10.1371/journal.pone.0209114

**Published:** 2018-12-20

**Authors:** Katherine B. Rucinski, Kimberly A. Powers, Sheree R. Schwartz, Brian W. Pence, Benjamin H. Chi, Vivian Black, Helen Rees, Audrey E. Pettifor

**Affiliations:** 1 Department of Epidemiology, University of North Carolina Gillings School of Global Public Health, Chapel Hill, NC, United States of America; 2 Wits Reproductive Health and HIV Institute, University of the Witwatersrand, Johannesburg, South Africa; 3 Department of Epidemiology, Johns Hopkins Bloomberg School of Public Health, Baltimore, MD, United States of America; 4 Department of Obstetrics and Gynecology, University of North Carolina School of Medicine, Chapel Hill, NC, United States of America; 5 Clinical Microbiology and Infectious Diseases, Faculty of Health Sciences, University of the Witwatersrand, Johannesburg, South Africa; University of Washington, UNITED STATES

## Abstract

**Objectives:**

Fertility intentions and contraceptive use are often not assessed in the context of clinical HIV care, representing a possible programming gap if women’s family planning needs change over time. We aimed to identify longitudinal patterns of unmet need for contraception over a 12-month period among women living with HIV taking antiretroviral therapy (ART).

**Study design:**

850 non-pregnant, HIV-positive women aged 18–35 on or initiating ART in Johannesburg, South Africa, were enrolled into a prospective cohort study in 2009–2010. Fertility intentions and contraceptive use were assessed during routine HIV care visits via an interviewer-administered questionnaire, and women were referred for on-site contraceptive counseling. We used group-based trajectory modeling to identify patterns of unmet need for contraception over 12 months, first in the entire population and then in a subset of recent ART initiators.

**Results:**

In the full population we identified four patterns of unmet need for contraception over one year. Half of the enrolled women were predicted to have a consistently high probability of unmet need, 22.9% a consistently low probability, 16.7% a decreasing probability, and 10.4% an increasing probability over time. Contraceptive method discontinuation and rapidly changing fertility intentions were the primary drivers of changing (increasing or decreasing) unmet need over follow-up. Results were similar in recent ART initiators.

**Conclusions:**

Half of women were estimated to have a high probability of unmet need that persisted over time, and more than a quarter were estimated to experience patterns of changing unmet need over 12 months. Family planning needs should be assessed more regularly in HIV-positive women to prevent unintended pregnancies and support safer conception among women trying to conceive.

## Introduction

Prevention of unintended pregnancy through reliable contraceptive methods improves women’s health and reduces both maternal and infant mortality among women living with HIV [1,2]. Prevention of unintended pregnancy is also an effective strategy to reduce mother-to-child HIV transmission [3], and is the second prong of WHO’s four-pronged framework for prevention of mother-to-child transmission (PMTCT) [4]. For these reasons and to ensure women’s reproductive rights, South African national contraception and family planning guidelines promote the availability of accessible and comprehensive contraceptive services to meet the family planning needs of women living with HIV [5]. In practice, however, women living with HIV in South Africa still experience tremendous difficulties meeting their family planning needs [6–9].

Family planning needs of women living with HIV are likely to change over time, yet these changes may go undetected due to facility- and provider-level factors that preclude providers from routinely assessing fertility preferences and contraceptive use in the context of HIV clinical care. Comprehensive programs to reduce unmet need for contraception in HIV-positive women, broadly defined as a discrepancy between expressed fertility preferences and contraceptive use [10], have traditionally focused on improving availability of services, increasing education and information dissemination around contraceptive use, and strengthening contraceptive counseling at the provider level [7,11–16]. However, even in settings where these enhanced family planning services are offered within HIV clinics, women may be reluctant to seek contraceptive counseling, and health-facility factors such as high patient volume and limited number of providers can affect patient-provider interaction time and quality of care [17–19]. To the extent that fertility intentions and contraceptive use are dynamic, this presents a missed opportunity for intervention.

Delineating patterns of unmet contraceptive need among women living with HIV could help providers better understand the extent to which the risk for unintended pregnancy is likely to change over time. Furthermore, characterization of temporal trends or predictors of unmet need for contraception could inform more targeted screening practices within integrated reproductive health and antiretroviral therapy (ART) services, including both contraception provision and safer conception interventions. We assessed longitudinal patterns of unmet need for contraception over a 12-month period within a cohort of women living with HIV taking ART in South Africa.

## Methods

### Study setting, population and design

Data were prospectively collected from four public-sector HIV outpatient clinics in inner city Johannesburg between 2009 and 2011 as part of a larger study to estimate the 12-month incidence of pregnancy among women living with HIV on ART. Full study procedures and eligibility criteria have been previously described [8,17,20]. In brief, all HIV-positive, non-pregnant, sexually active women between the ages of 18–35 on or initiating ART were eligible for participation if they had not given birth in the last three months, were not breastfeeding, had not had a previous tubal ligation, hysterectomy, bi-lateral oophorectomy, and had not received a diagnosis of permanent infertility. Pregnancy was determined using urine-based tests administered by study staff (One Step hCG Urine Pregnancy Test, Atlas Link Technology, Beijing) and confirmed through repeat testing. ART regimen information, CD4 cell count and viral load data were confirmed through medical record review and pharmacy and laboratory records. After providing written informed consent, women meeting eligibility criteria completed an interviewer-administered questionnaire to assess demographic and behavioral characteristics including sexual risk behaviors, fertility history and intentions, and contraceptive use. Women were referred for antenatal care, on-site contraceptive counseling, or other services as needed.

### Outcome definition

Our outcome of interest was unmet need for contraception (yes/no), which we assessed at the time of each routine HIV care visit. Our assessment was guided by the algorithm for population-level unmet need for contraception as proposed by Bradley et al. [21], which we used as a framework for determining each woman’s individual-level unmet need during study visits (see S1 Questionnaire). Specifically, women were considered to have unmet need for contraception at a given study visit if they reported 1) being married, living with a partner, or being sexually active in the three months preceding study enrollment; 2) not currently trying to become pregnant (at time of interview); and 3) not currently using oral contraceptives, injectable, a hormonal implant or an intrauterine device. Women who met the first two criteria and reported condom use as their primary family planning method were considered to have unmet need, as rates of unintended pregnancy are comparable among women who do not use family planning methods and among those who report condom use alone [8,22].

We assessed unmet need for contraception at monthly intervals after enrollment, corresponding to the routine care visits, for a total of 12 time points. In cases where a woman had two routine care visits within a given month, we employed a two-stage process for measurement selection. If another visit occurred in the subsequent interval, we retained only the first measurement for the month in question. If, however, no visit occurred in the next interval, we retained both measurements for the month in question provided they occurred at least 21 days apart, and assigned the second value forward to the next interval. This process accounted for the heterogeneity in clinic visit frequency among women, minimized missing data, and reflected any conflicting, individual measurement within a given interval. Women were censored at pregnancy, loss to follow-up, death, or the completion of 12 months of follow-up.

The original study protocol was approved by the University of the Witwatersrand Human Research Ethics Committee, Johannesburg, South Africa. Written informed consent was obtained from all participants. The University of North Carolina at Chapel Hill Institutional Review Board also approved the secondary analysis presented here.

### Statistical analysis

We used group-based trajectory modeling to identify distinct longitudinal patterns of unmet need for contraception over the 12-month period [23,24]. Group-based trajectory modeling is an application of finite mixture modeling designed to identify clusters of individuals who follow a similar trajectory for a given outcome over time [24]. We first fit a series of predictor-free trajectory models to identify the optimal number of trajectories describing the population patterns of unmet need for contraception, fitting models with 2, 3, 4, or 5 trajectory groups to allow for heterogeneity while ensuring interpretability. Given the dichotomous nature of the outcome, we specified a logit link and binomial distribution, and we decided *a priori* to consider both quadratic and cubic specifications of each model to enable flexible trajectory shapes. We chose the optimal model based on optimum Bayesian information criterion (BIC) using Jeffreys’s scale of evidence for Bayes factors [25,26].

As unmet need for contraception is a function of both fertility intentions and contraceptive use, changes in unmet need may reflect changes in fertility intentions, contraceptive use, or both. Therefore, to delineate changes in the separate phenomena contributing to the composite outcome of unmet need for contraception, we conducted a descriptive analysis in which we graphically characterized both fertility intentions and contraceptive use over the study period. At each interval we assigned women to one of four mutually exclusive categories:1) those who had unmet need according to the definition above; 2) those who were married, living with a partner, or sexually active and using a reliable method of contraception to prevent pregnancy; 3) those who were married, living with a partner, or sexually active and trying to conceive, and 4) those who were not married, living with a partner, or sexually active. We conducted this descriptive analysis overall and by trajectory group (assigned on the basis of the maximum posterior probability of group membership [23,24]).

To identify predictors of trajectory group membership, we first constructed a categorical measure for group membership based on the final predictor-free model, assigning each woman to a group based on her maximum posterior membership probability in each model [23,24]. After assigning women to groups, we conducted descriptive, within-group analyses of clinical and behavioral features assessed at enrollment. These features, which we identified through a literature review of factors associated with unmet need for contraception, were related to demographics, measures of fertility history and contraceptive use, partner-associated characteristics, measures of patient/provider interaction and HIV-related clinical outcomes. We then fit a multinomial logistic regression model with the categorical measure for group membership as the outcome and the aforementioned predictors of interest as explanatory variables.

In a second stage of the predictor analysis, we constructed a new group-based trajectory model for unmet need for contraception, retaining all predictors that were found to be significant (α = 0.1) for at least one trajectory group comparison in the first stage [23]. We included this second stage to ensure that our coefficients and standard errors properly accounted for covariance between parameter estimates and the probabilities of membership in each trajectory group [23].

We conducted all analyses in the full cohort and repeated them in a subgroup of women initiating ART within 3 months of enrollment, given the likelihood that fertility intentions may change rapidly immediately after ART initiation [27–29], as well as the distinctness of ART initiation as a clearly identifiable developmental origin and intervention point. In sensitivity analyses for both the full cohort and recent initiators, we re-estimated trajectories using quarterly interval assessments (every three months). All models were fit using Proc Traj (https://www.andrew.cmu.edu/user/bjones/index.htm), a free downloadable SAS add-on package (SAS, version 9.4, Cary, NC).

## Results

### Study population characteristics

A total of 850 women were enrolled and followed for up to 12 months. Over the 12-month period, 149 women (17.5%) became pregnant and contributed a median of 6.2 months (IQR 4, 9) of follow-up before they were censored at time of pregnancy. Among the 701 women who did not become pregnant, median follow-up time was 11.4 months (IQR 9, 12), with 28 (4.0%) not returning to care after their initial study visit. Median time between follow-up visits was 1.7 months (IQR 1, 2) for women who became pregnant and 1.8 months (IQR 1, 2) for those who did not. No deaths were reported over the study period.

Women were on average 30 years of age (IQR 27, 33), and the majority were in a relationship (789/850, 92.8%) (Table 1). Most women (760/850, 89.4%) had previously been pregnant, with a median time since last pregnancy of 48 months (IQR 20, 96). The median time since HIV diagnosis was 24 months (IQR 12, 48), and median time since ART initiation was 13 months (IQR 5, 24).

**Table 1:** Characteristics at enrollment of 850 women living with HIV taking ART in Johannesburg by ART initiation status, 2009–2011.

	Overall N = 850		ART Experienced* N = 693		Recent ART Initiators^†^ N = 157	
	Median	IQR	Median	IQR	Median	IQR
**Age (years)**	30.4	27–33	30.5	28–33	30.0	26–32
**Monthly income (ZAR)**	2000.0	1000–3480	2000.0	1000–3500	1700.0	1000–3000
**No. living children**	1.0	1–2	1.0	1–2	1.0	0–2
**Months since last pregnancy**	48.0	20–96	48.0	18–84	72.0	26–96
**Months since HIV diagnosis**	24.0	12–48	28.0	15–48	11.0	3–24
**CD4 count at enrollment (cells/μl**)	312.0	178–462	356.0	242–510	149.0	87–179
**Months on ART**	13.0	5–24	16.0	9–29	1.0	0–2
	**n**	**%**	**n**	**%**	**n**	**%**
**Education completed**						
None-Grade 10	55	6.5	44	6.4	11	7.0
Grade 11-Grade 12	671	78.9	542	78.2	129	82.2
Post-grad degree or certificate	124	14.6	107	15.4	17	10.8
**Employed**	510	60.0	427	61.6	83	52.9
**Married/cohabitating with a partner**	378	44.5	300	43.3	78	49.7
**In a relationship**	789	92.8	655	94.5	134	85.4
**Sexually active, last three months**	754	89.4	627	91.0	127	82.5
**Partner HIV status**^‡^						
Negative	200	25.3	165	25.2	35	25.7
Positive	312	39.4	260	39.7	52	38.2
Unknown	279	35.3	230	35.1	49	36.0
**Ever pregnant**	760	89.4	630	90.9	130	82.8
**Pregnant at HIV diagnosis**	271	31.9	248	35.8	23	14.7
**Trying to conceive, currently**	105	12.4	83	12.0	22	14.0
**Trying to conceive, next year**	396	46.6	299	43.2	97	61.8
**Taking hormonal contraception**	224	26.4	203	29.3	21	13.4
**Using condoms for family planning**	410	48.2	325	46.9	85	54.1
**Unmet need for contraception**	500	58.8	415	59.9	85	54.1

Abbreviations; IQR: Interquartile Range, ART: Antiretroviral therapy

^*^ On ART >3 months at study entry

^**†**^ Initiated ART within three months of study entry

^‡^n = 791

Of the 850 women in the full cohort, 157 (18.5%) had initiated ART within three months of study enrollment (“recent initiators”) and 693 (81.5%) had initiated ART more than three months prior to study enrollment (“ART experienced”). Demographics, behavioral characteristics and clinical characteristics differed by ART experience. Compared to ART-experienced women, a larger proportion of recent initiators reported at enrollment that they planned to become pregnant in the next 12 months (97/157 (61.8%) vs. 299/693 (43.2%)) and a smaller proportion were using hormonal contraception (21/157 (13.4%) vs. 203/693 (29.3%)) to prevent pregnancy.

Unmet need was assessed at study enrollment in both the full cohort and in recent ART initiators. Of the 850 women included in the full cohort, 500 (58.8%) had unmet need. The proportion of women with unmet need was similar among recent initiators of ART compared to women who were ART experienced (54.1% vs. 59.9%).

### Longitudinal assessment of unmet need for contraception

**Fig 1:**
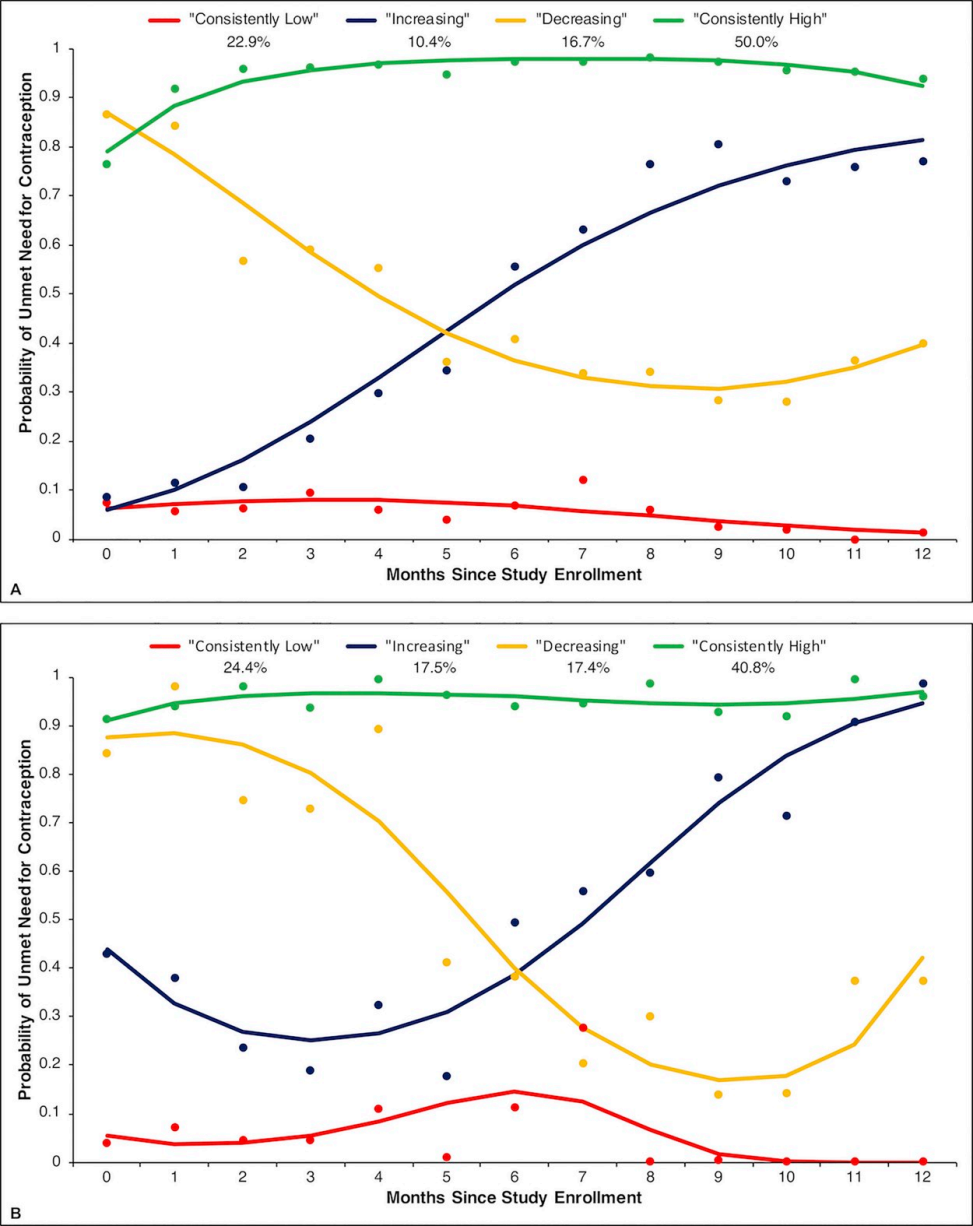
Predicted trajectories of unmet need for contraception among women living with HIV taking ART in Johannesburg, 2009–2011. Panel A represents predicted trajectories for the four-group quadratic model estimated for the full cohort (N = 850). Panel B represents predicted trajectories for the four-group cubic model estimated for recent initiators of ART (N = 157). Dots represent the observed proportion of women with unmet need for contraception among those assigned to a given trajectory group on the basis of their maximum posterior group membership probability. Curves represent the proportion of women with unmet need for contraception as estimated by the model for a given trajectory group.

A four-group quadratic model was selected as the optimal predictor-free model to identify patterns of unmet need over 12 months (Fig 1). Half (50.0%) of the cohort was predicted to have a consistently high probability of unmet need (predicted probability of unmet need between 79.0% and 98.0%) over the study period. Nearly a quarter (22.9%) were predicted to have a consistently low probability of unmet need (1.2%-8.0% probability of unmet need over time). Additionally, 16.7% had a steadily decreasing probability of unmet need (from 86.9% at month 0 to 30.8% at month 9) and 10.4% exhibited a sharp increase in this probability (from 6.0% at month 0 to 81.4% at month 12). Based on these trends for unmet need, we qualitatively described these groups as “consistently high,” “consistently low,” “decreasing,” and “increasing,” respectively.

In the sub-analysis of recent ART initiators (n = 157), a four-group, predictor-free cubic model was selected to identify patterns of unmet need (Fig 1). Similar to the model selected for the full cohort, nearly half (40.8%) of recent initiators were predicted to have a consistently high probability of unmet need (predicted probability of unmet need between 91.1% and 96.9% throughout). Women who were predicted to have a consistently low probability of unmet need (between 0.0% and 14.6% across the 12 months) accounted for 24.4% of recent initiators. Nearly one-fifth (17.5%) of recent initiators were predicted to have a slowly increasing probability of unmet need (from 25.1% at month 3 to 94.5% at month 12), and a nearly identical proportion (17.4%) were predicted to have a decreasing probability of unmet need (from 88.3% at month 1 to 16.9% at month 9).

In sensitivity analyses where we re-estimated trajectories using quarterly intervals assessments, results were generally similar (S1 Fig).

### Fertility intentions and contraceptive use over time

**Fig 2:**
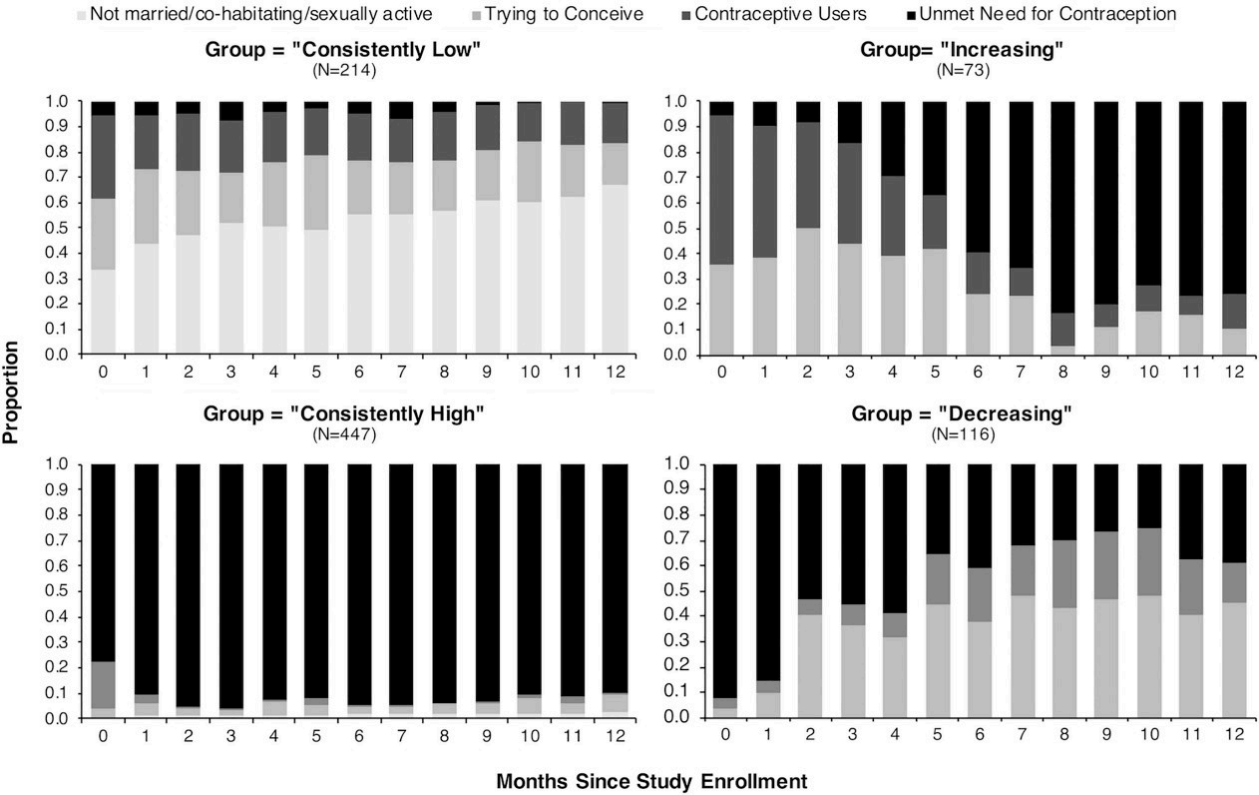
Fertility intentions and contraceptive use over time, stratified by assigned trajectory group based on each participant’s highest posterior group-membership probability for the full cohort (N = 850). At each monthly assessment women were either 1) not married, co-habitating, or sexually active, 2) trying to conceive, 3) using a reliable method of contraception to prevent pregnancy (contraceptive users) and 4) not using a reliable method of contraception to prevent pregnancy (unmet need for contraception).

Temporal patterns in the two distinct contributors to unmet need–fertility intentions and contraceptive use–varied dramatically across trajectory groups (Fig 2). In those who followed an increasing trajectory of unmet need (based on assignment to the increasing group using a maximum posterior membership probability), the proportion trying to conceive decreased over time (from 35.6% in month 0 to 10.3% in month 12), as did the proportion using reliable contraception to prevent pregnancy (from 61.6% in month 0 to 13.8% in month 12). In those who followed a decreasing trajectory of unmet need, the proportion trying to conceive increased over time (from 3.5% at month 0 to 45.5% at month 12) and the proportion using reliable contraception to prevent pregnancy also increased (between 4.3% and 16.9% over the 12 months). For women who followed trajectories of unmet need that were relatively constant (i.e. consistently low or consistently high), we observed little change in fertility intentions and contraceptive use over the 12 months. Results were similar in recent initiators.

### Predictors of trajectory group membership

Our multinomial logistic regression analysis allowed comparison of predictors for each of the four trajectory groups relative to each other group. For simplicity, we focus here on the two main comparisons of greatest apparent clinical relevance. Specifically, based on our observation of similar “starting points” for the consistently high and decreasing trajectories (and for the consistently low and increasing trajectories), we describe predictors associated with membership in the decreasing group (compared to the consistently high group) and those associated with membership in the increasing group (compared to the consistently low group), as found using the two-stage process outlined above. Compared to the consistently high unmet need group, those in the decreasing group were likely to have fewer children (OR 0.4, 95% CI 0.3, 0.5 per one-unit increase in the categorical variable describing number of living children) and were more likely to have a partner who desired a/another child (OR 4.2 95% CI 1.6, 10.9) (Table 2). Compared to the consistently low group, women in the increasing group were more likely to have reported a problem with their contraceptive method (i.e., heavy bleeding, irregular bleeding and spotting, amenorrhea, rash, or thrush as well as common side effects such as weight gain or headaches) (OR 3.0, 95% CI 1.4, 6.6) (Table 3).

**Table 2:** Odds ratios (95% confidence intervals) associated with membership in the decreasing unmet need trajectory group (compared to membership in the consistently high unmet need group) (N = 850).

	OR (95% CI)			
Characteristic	Consistently High	Decreasing	Increasing	Consistently Low
Number of living children^**†**^	**1**	0.4 (0.3, 0.5)	0.6 (0.4, 0.9)	0.6 (0.4, 0.7)
Employed	**1**	1.7 (0.9, 3.0)	1.9 (0.8, 4.4)	1.5 (0.9, 2.7)
Main partner desires a/another child	**1**	4.2 (1.6, 10.9)	0.5 (0.1, 2.9)	0.8 (0.5, 1.2)
Problems with contraceptive method^‡^	**1**	0.7 (0.3, 1.4)	1.9 (1.0, 3.7)	0.7 (0.4, 1.3)

Abbreviations; OR: Odds Ratio, CI: Confidence Interval

^**†**^ Per increase in category (corresponding to an increase in total number of children), with 1 = 0 children, 2 = 1 child, 3≥2 children

^‡^ Includes heavy bleeding, irregular bleeding and spotting, amenorrhea, and rash or thrush as well as common side effects such as weight gain or headaches

**Table 3:** Odds ratios (95% confidence intervals) associated with membership in the increasing unmet need trajectory group (compared to membership in the consistently low unmet need group) (N = 850).

	OR (95% CI)			
Characteristic	Consistently Low	Increasing	Decreasing	Consistently High
Number of living children^**†**^	**1**	1.2 (0.8, 1.9)	0.7 (0.5, 1.0)	2.9 (1.5, 2.6)
Employed	**1**	1.1 (0.6, 2.2)	2.1 (1.2, 3.8)	1.2 (0.8, 1.8)
Main partner desires a/another child	**1**	1.3 (0.5, 3.4)	2.0 (0.8, 4.9)	0.6 (0.3, 1.0)
Problems with contraceptive method^‡^	**1**	3.0 (1.4, 6.6)	1.2 (0.5, 2.8)	1.3 (0.7, 2.4)

Abbreviations; OR: Odds Ratio, CI: Confidence Interval

^**†**^ Per increase in category (corresponding to an increase in total number of children), with 1 = 0 children, 2 = 1 child, 3≥2 children

^‡^ Includes heavy bleeding, irregular bleeding and spotting, amenorrhea, and rash or thrush as well as common side effects such as weight gain or headaches

In analyses confined to recent initiators, we were unable to identify meaningful predictors of group membership in stage one due to the limited number of women assigned to each trajectory group, and models did not converge in stage two. Additional descriptive profiles of trajectory group membership for both the full cohort and recent initiators are reported in S1 and S2 Tables.

## Discussion

Changes in fertility intentions following ART initiation, coupled with improvements in fecundity [22,30] and renewed sexual desire and greater coital frequency [27] are thought to contribute to increasing incidence of pregnancies in women living with HIV after starting ART in the absence of compensatory contraceptive use. In this study, we demonstrated that women living with HIV on ART in South Africa have a high probability of unmet need for contraception, and that rapidly changing fertility intentions and decisions about contraceptive use contribute to changing unmet need in a non-trivial subset of women.

Half of women in our full cohort were predicted to have a consistently high probability of unmet need that persisted over time. Approximately one in ten women in our full cohort and almost 20% of those recently initiating ART were predicted to have unmet contraceptive need that increased over time, indicating they were at higher risk of unintended pregnancy as time under observation increased. Notably, among women with increasing unmet contraceptive need, few were observed to have unmet need at study enrollment (S1 Table). Our findings are compatible with previous research showing a single assessment of contraceptive need is insufficient in identifying those at high risk for unintended pregnancy, even within a 12-month period [8].

Consistent with other estimates of contraceptive use in South Africa, few women in our study were using a reliable method of contraception to prevent pregnancy at enrollment. Low uptake of hormonal contraception may reflect fear of side effects or health risks (e.g., heavy menstrual bleeding, irregular bleeding, bleeding between periods, lower abdominal pain), which are strongly associated with contraceptive discontinuation or switching in HIV-positive women [31,32,33]. Limited uptake or method discontinuation may also reflect structural barriers such as long clinic wait times [17,18] or other unknown factors, as well as additional fears around side effects resulting from ART/contraceptive drug interactions [34]. However, only one woman in our study reported discontinuing her method for this reason. Compared to women in our study who followed a trajectory of consistently low unmet contraceptive need, those with increasing unmet need were significantly more likely to report problems or side effects with their method of contraception. Furthermore, a significant proportion of those who followed an increasing trajectory of unmet need discontinued their method of contraception over the 12-month period without a corresponding change in fertility intentions.

Changes in unmet need for contraception also reflect changes in fertility intentions. We found that among women with a decreasing unmet need for contraception over follow-up, the primary driver of reduced unmet need was increasing fertility intentions, not an increase in uptake of contraception. Previous studies have found that fertility intentions increase rapidly in the period following ART initiation [27–29], though evidence is limited. Increasing fertility intentions among women on ART may also reflect partner fertility desires, and we found that women with a decreasing unmet contraceptive need were more likely to report a partner who desired a/another child. To the extent that fertility intentions reflect decisions around childbearing in both partners, comprehensive screening of family planning needs that includes asking women about their partner’s fertility intentions may offer additional opportunities to identify women who require safer conception counseling.

We observed that family planning needs of women living with HIV changed frequently over time in this South African setting, yet in clinical practice fertility intentions and contraceptive use were infrequently assessed. While current national service delivery guidelines promote the integration of sexual and reproductive health services with HIV prevention, care and treatment in South Africa [5], implementation of these guidelines in clinical practice has been limited [35,36]. Women in our study, for example, received on-site referrals for contraceptive counseling that technically constitute integrated services. However, providers were frequently located in different rooms or wards, contributing to long queues and lengthy wait times. Preliminary results from the Tsepamo Study in Botswana indicating an increased risk for neural tube defects among women taking dolutegravir at conception have spurred further guidance around increasing contraceptive uptake for women initiating ART [37]. However, the extent to which this additional guidance will ultimately streamline delivery of family planning services is yet to be determined.

Routine HIV clinical care visits represent a potential opportunity to regularly assess family planning needs, particularly among women on ART who may be unaware that their fertility will increase once started on ART [22,30,38]. Furthermore, regularly assessing the family planning needs of women who have trouble achieving viral suppression is critical for both prevention of horizontal transmission to HIV-uninfected partners and PMTCT. Previous studies have suggested that multidimensional measures of fertility intentions (e.g., measures that incorporate questions around childbearing preferences, feelings about a pregnancy/child, and perceived consequence of a pregnancy/child) are needed to reliably determine women’s family planning needs [39,40]. However, operationalizing more complex tools within the context of high patient volume and limited provider resources may pose further challenges. More succinct measures of fertility intentions, such as those used to inform this study’s assessment of unmet need, are demonstrably reliable in predicting pregnancy outcomes [8,17,41] and may be more readily integrated into clinical practice to evaluate the immediate family planning needs of women living with HIV. In one high-volume facility in Malawi, integration of a short family planning assessment using an electronic medical record-based tool prompted healthcare workers to more routinely offer contraceptives during HIV care visits, increasing contraceptive prevalence over a four-year period [42].

Our study is not without limitations. First, nearly half of women in this analysis reported condoms as their primary method of family planning at enrollment but were considered to have unmet need for contraception. As some of these women may have used condoms consistently and correctly, we may have overestimated the total number of women in our cohort with unmet need. However, women in this study who reported using condoms had nearly identical pregnancy rates as women who reported using no contraceptive method [8], suggesting that the majority of women using condoms were not doing so effectively enough to prevent pregnancy. Second, all behavioral outcomes were measured by self-report and are subject to potential misclassification; however, questionnaires were administered in private by trained study staff who developed a strong rapport with the participants to minimize social desirability bias. Third, data included in this study predate South African national service delivery guidelines for integrated family planning and HIV care and earlier thresholds for treatment initiation. At the time of study enrollment, non-pregnant women were only eligible for lifelong ART initiation with a CD4 count ≤200 cells/μl or WHO clinical stage 4 diagnosis [43], and trajectories of unmet need (and predictors thereof) may differ in the current era. We were also unable to confirm the original indication for treatment among women who initiated ART prior to enrolling in this study, and trajectories of unmet need may also differ among women who initiated ART through PMTCT compared to women who initiated ART for their own health. However, we saw very little difference in CD4 count by trajectory group overall, and trajectories were largely similar between the whole cohort and the subgroup of recent ART initiators who were known to have started treatment outside of pregnancy. Furthermore, operational standards around the assessment of fertility intentions and the provision of effective methods of contraception (e.g., long-acting reversible contraception) within the ART clinic setting remain relatively unchanged in current clinical practice.

## Conclusions

We have demonstrated that in this South African setting, a high proportion of women living with HIV experience a persistently high probability of unmet need for contraception. We have also shown that the family planning needs of women living with HIV may change rapidly, even over a 12-month period. As more and more women initiate ART through South Africa’s universal testing and treatment initiative, routine screening of fertility intentions combined with rapid service referral for reliable contraception and safer conception methods will be critical in ensuring that women living with HIV are able to meet their reproductive needs.

## Supporting information

S1 Questionnaire Fertility intentions and contraceptive use assessment items. (PDF)

S1 Fig Predicted trajectories of unmet need for contraception using quarterly measurements among women living with HIV taking ART in Johannesburg, 2009–2011. Figure that illustrates trajectories of unmet need for contraception estimated using quarterly assessments for A) the full cohort (N = 850) and B) recent ART initiators (N = 157).
(TIFF)

S1 Table Characteristics of Women Assigned to “Consistently Low,” “Increasing,” “Decreasing,” and “Consistently High” Unmet Need Trajectory Groups using a Maximum-Probability Assignment Rule (N = 850). (DOCX)

S2 Table Characteristics of Recent ART Initiators Assigned to “Consistently Low,” “Increasing,” “Decreasing,” and “Consistently High” Unmet Need Trajectory Groups using a Maximum-Probability Assignment Rule (N = 157). (DOCX)

S1 Dataset (ZIP)
