# Supplementary material for: Longitudinal patterns of unmet need for contraception among women living with HIV on antiretroviral therapy in South Africa
Source: PLoS One. 2018 Dec 20;13(12):e0209114. doi: 10.1371/journal.pone.0209114 (PMC6301780; doi:10.1371/journal.pone.0209114)
Supplement: S1 Questionnaire — (PDF) [file pone.0209114.s001.pdf]

S1 Questionnaire

A comprehensive assessment of fertility intentions and contraceptive use was conducted at Enrollment (Questionnaire A). A rapid assessment was conducted at each Follow-Up visit (Questionnaire B). Our primary measure of fertility intentions in this study was assessed using the question "Are you currently trying to get pregnant?", which was asked both at Enrollment (Q101) and during Follow-up (Q2).

A. Fertility Intentions and Contraceptive Use Comprehensive Assessment. *Questions asked at Enrollment*

SECTION: FERTILITY INTENTIONS		
**Q101.**	**Are you currently trying to get pregnant?**	☐ **Yes → Q102**<br>☐ **No → Q103**<br>☐ **Not sure → Q103**
**Q102.**	**How long have you been trying to have a baby this time? <u>You can tell me in months or years.</u>**	☐ **Years ……….→ Q201**<br>☐ **Months ……….→ Q201**
**Q103.**	**Thinking about the next year, do you plan to become pregnant <u>in the next 12 months</u> (1 year)?**	☐ **Yes → Q201**<br>☐ **No → Q104**<br>☐ **Not sure → Q104**
**Q104.**	**Thinking further ahead to the future, do you think that <u>someday</u> you may try to become pregnant?**	☐ **Yes → Q201**<br>☐ **No → Q201**<br>☐ **Not sure → Q201**

SECTION: SEXUAL BEHAVIOR AND CONTRACEPTIVE USE		
**Q201.**	**Are you currently using any method of contraception for family planning?**	☐ **Yes → Q202**<br>☐ **No → Q209**
**Q202.**	**What method(s) of family planning are you currently using?**<br>*Do not read responses. Mark all that apply.*<br>*Probe for more answers.*	☐ **Pill** (Oral Contraceptives)<br>o Combined Pill (COC, E+P)<br>o Progestin-only Pill<br>o Unknown Pill<br>☐ **Male Condom**<br>☐ **Female Condom**<br>☐ **Injectable**

		○ Depo-Provera or Petogen<br>○ Nuristerate<br>○ Norigynon<br>○ Type unknown<br>☐ **IUD** (Intrauterine Device, e.g. Mirena)<br>☐ **Spermicide**<br>☐ **Breastfeeding/ Lactational Amenorrhea Method (LAM)**<br>☐ **Implant**<br>☐ **Partner was sterilized/ vasectomy**<br>☐ **Emergency Contraception**<br>☐ **Other______________________**<br>☐ **Nothing**
**Q203.**	**How long have you been using this (these) method(s) of contraception for family planning? <u>You can respond in months or years.</u>**	☐ **Years ……….**<br>☐ **Months ……….**
**Q204.**	**Have you been having any problems with the method?**	☐ **Yes → Q205**<br>☐ **No → Q208**
**Q205.**	**What is the biggest problem that you have been experiencing with your current method?**<br>*Do not read responses. Mark all that apply.*	☐ **Heavy bleeding**<br>☐ **Irregular spotting or bleeding**<br>☐ **No period, amenorrhea**<br>☐ **Wet vagina**<br>☐ **Weight gain**<br>☐ **Headaches**<br>☐ **Abdominal Pain / Cramping**<br>☐ **Partner does not like or agree to use current method**<br>☐ **Difficult to remember to take the pill everyday**<br>☐ **Difficult to regularly get injections**<br>☐ **Difficult to get prescription filled**<br>☐ **Difficult to buy condoms**<br>☐ **Other______________________**

**Q206.**	**What other problems are you experiencing with your current method?**<br>*Do not read responses. Mark all that apply.*<br>*Probe for more answers.*	☐ **Heavy bleeding**<br>☐ **Irregular spotting or bleeding**<br>☐ **No period, amenorrhea**<br>☐ **Wet vagina**<br>☐ **Weight gain**<br>☐ **Headaches**<br>☐ **Abdominal Pain / Cramping**<br>☐ **Partner does not like or agree to use current method**<br>☐ **Difficult to remember to take the pill everyday**<br>☐ **Difficult to regularly get injections**<br>☐ **Difficult to get prescription filled**<br>☐ **Difficult to buy condoms**<br>☐ **Other________________________**
**Q207.**	**Have you talked to your nurse or doctor about the problems that you just told me about?**	☐ **Yes**<br>☐ **No**<br>☐ **Don't know**
**Q208.**	**Overall how satisfied are you with your current contraceptive / family planning method? Would you say that you are very satisfied, somewhat satisfied or dissatisfied?**	☐ **Very satisfied**<br>☐ **Somewhat satisfied**<br>☐ **Dissatisfied**<br>☐ **Don't know**
**Q209.**	**In the next month, do you plan on continuing with the same method of family planning, start using a new method or stop using contraception?**<br>*Should also be asked of women not using a method of contraception to assess whether they plan to start a new method.*	☐ **Continue with same method → Q212**<br>☐ **Start new method → Q211**<br>☐ **Stop using method → Q210**
**Q210.**	**Why do you plan to quit the method?**<br>*Remember, do not read responses.*<br>*Mark all that apply. Probe.*	☐ **Trying to get pregnant**<br>☐ **Not sexually active**<br>☐ **Do not think I can get pregnant**<br>☐ **No period, amenorrhea**<br>☐ **Partner does not approve of method**<br>☐ **Partner had a vasectomy**

		☐ **Side effects of method**<br>☐ **Method too difficult**<br>☐ **Heavy bleeding**<br>☐ **Irregular spotting or bleeding**<br>☐ **Method less effective with HIV medication**<br>☐ **Weight gain**
**<u>Skip to Q212</u>**		
**Q211.**	**What family planning method(s) do you plan to start using in the next month?**<br>*Do not read responses. Mark all that apply*	☐ **Pill** (Oral Contraceptives)<br>o Combined Pill (COC, E+P)<br>o Progestin-only Pill<br>o Unknown Pill<br>☐ **Male Condom**<br>☐ **Female Condom**<br>☐ **Injectable**<br>o Depo-Provera or Petogen<br>o Nuristerate<br>o Norigynon<br>o Type unknown<br>☐ **IUD** (Intrauterine Device, e.g. Mirena)<br>☐ **Spermicide**<br>☐ **Breastfeeding/ Lactational Amenorrhea Method (LAM)**<br>☐ **Implant**<br>☐ **Partner was sterilized/ vasectomy**<br>☐ **Emergency Contraception**<br>☐ **Other______________________**
**Q212.**	**The last time that you had sex with your main partner did you and your partner use a condom?**	☐ **Yes**<br>☐ **No**<br>☐ **Don't know**<br>☐ **No response**
**Q213.**	**During the past 12 months, how often have you and your main partner used a condom?**	☐ **Every time**<br>☐ **Almost every time**

	<u>**Did you use a condom every time, almost every time, sometimes or never?**</u>	☐ **Sometimes**<br>☐ **Never**<br>☐ **Don't know**<br>☐ **No response**
**Q214.**	**The last time that you had sex with a casual or nonregular partner did you and that partner use a condom?**	☐ **Yes**<br>☐ **No**<br>☐ **Don't know**<br>☐ **No response**<br>☐ **Not applicable – other partners were main partners at the time of intercourse**
**Q215.**	**During the past 12 months, how often have you and your non-regular partner(s) used a condom?**<br><u>**Did you use a condom every time, almost every time, sometimes or never?**</u>	☐ **Every time**<br>☐ **Almost every time**<br>☐ **Sometimes**<br>☐ **Never**<br>☐ **Don't know**<br>☐ **Not applicable**
**CHECK**	*Is the participant currently on family planning other than condoms? (See Q201 and Q202 for clarification)*<br>☐ **IF Yes → Q221**<br>☐ **IF NO → Q216**	
**Q216.**	**During the past 6 months, have you used any method of contraception for family planning <u>other than condoms?</u>**	☐ **Yes → Q217**<br>☐ **No → Q220**<br>☐ **No response → Q221**
**Q217.**	**What method(s) were you using?**<br>*Do not read responses.*<br>*Mark all that apply*	☐ **Pill** (Oral Contraceptives)<br>○ Combined Pill (COC, E+P)<br>○ Progestin-only Pill<br>○ Unknown Pill<br>☐ **Injectable**<br>○ Depo-Provera or Petogen<br>○ Nuristerate<br>○ Norigynon<br>○ Type unknown<br>☐ **IUD** (Intrauterine Device, e.g. Mirena)<br>☐ **Spermicide**

		☐ Breastfeeding/ Lactational Amenorrhea Method (LAM)<br>☐ Implant<br>☐ Partner was sterilized/ vasectomy<br>☐ Emergency Contraception<br>☐ Other_______________________<br>☐ Nothing
**Q218.**	**Why did you quit the method?**<br>*Remember, do not read responses.*<br>*Mark all that apply. Probe.*	☐ Trying to get pregnant<br>☐ Not sexually active<br>☐ Do not think I can get pregnant<br>☐ No period, amenorrhea<br>☐ Partner does not approve of method<br>☐ Partner had a vasectomy<br>☐ Side effects of method<br>☐ Method too difficult<br>☐ Heavy bleeding<br>☐ Irregular spotting or bleeding<br>☐ Method less effective with HIV medication<br>☐ Do not know which method to use<br>☐ Don't know
**Q219.**	**Does your doctor or nurse know that you are no longer using that method?**	☐ Yes<br>☐ No<br>☐ Don't know
**Q220.**	**What is the main reason that you are not using a method for family planning?**<br>*Note that if the participant is using condoms, ask them what is the main reason that they are not using a method for family planning other than condoms.*	☐ Trying to get pregnant<br>☐ Not sexually active<br>☐ Do not think I can get pregnant<br>☐ No period, amenorrhea<br>☐ Partner does not approve of method<br>☐ Partner had a vasectomy<br>☐ Side effects of method<br>☐ Method too difficult<br>☐ Heavy bleeding<br>☐ Irregular spotting or bleeding

		☐ **Method less effective with HIV medication**<br>☐ **Do not know which method to use**<br>☐ **Not necessary, using condoms**<br>☐ **Other______________________**<br>☐ **Don't know**
**Q221.**	**Since you started ARVS, has your nurse or doctor talked to you about any of the following family planning methods?**<br>*READ ALL RESPONSES. Mark all that apply.*	**Yes No**<br>**Oral contraceptives or pills** ☐ ☐<br>**Injectable contraceptives** ☐ ☐<br>(Depo-Provera, Petogen or Nuristerate)<br>**Male Condoms** ☐ ☐<br>**Female Condoms** ☐ ☐<br>**Sterilization** ☐ ☐<br>**Other___________________**
**Q222.**	**What clinic do you currently go to for your family planning/contraceptive care?**	☐ **Name of Clinic___________ → Q223**<br>☐ **None → Q225**
**Q223.**	**Does the nurse or doctor at the other clinic know that you are HIV+?**	☐ **Yes → Q224**<br>☐ **No → Q225**<br>☐ **Don't know → Q225**
**Q224.**	**Is the other health provider aware of what HIV medicines you are currently taking?**	☐ **Yes**<br>☐ **No**<br>☐ **Don't know**
**Q225.**	**Have you heard about any family planning methods that may not work well to prevent pregnancy if you are also taking ARVs?**	☐ **Yes → Q226**<br>☐ **No → Q301**<br>☐ **Don't know → Q301**
**Q226.**	**What family planning methods have you heard do not work as well to prevent pregnancy when you are also on HIV treatment?**<br>*Mark all that are mentioned.*<br>*Note that Nordette and Triphasil are both classified as combined pills or COCs*	☐ **Pill** (Oral Contraceptives)<br>o Combined Pill (COC, E+P)<br>o Progestin-only Pill<br>o Unknown Pill<br>☐ **Injectable**<br>o Depo-Provera or Petogen

		○ Nuristerate<br>○ Norigynon<br>○ Type unknown<br>☐ **IUD** (Intrauterine Device, e.g. Mirena)<br>☐ **Spermicide**<br>☐ **Breastfeeding/ Lactational Amenorrhea Method (LAM)**<br>☐ **Implant**<br>☐ **Other______________________**<br>☐ **Don't know**

**SECTION: FERTILITY DESIRES**		
**Q301.**	**Have you and your partner talked about whether or not you would like to have more children?**	☐ **Yes**<br>☐ **No**<br>☐ **Don't know**
Sometimes people do not intend or plan on having more children, but they may still want or desire to have more children.		
**Q302.**	**Do you think that your partner wishes he could have more children?**	☐ **Yes → Q303**<br>☐ **No → Q304**<br>☐ **Don't know → Q304**
**Q303.**	**How important do you think it is to your partner that you have more children?**<br>**Would you say that it is very important to him, somewhat important or not important?**	☐ **Very important**<br>☐ **Somewhat important**<br>☐ **Not important**<br>☐ **Don't know**
**Q304.**	**Do you personally wish that you could have more children?**	☐ **Yes → Q305**<br>☐ **No → END**<br>☐ **Don't know → END**
**Q305.**	**How many more children would you like to have if you could choose?**	☐ **Number of additional desired……….**<br>☐ **None**<br>☐ **Don't know**

B. **Fertility Intentions and Contraceptive Use Rapid Assessment.** *Questions asked during Follow-Up*		
**Q1.**	**Are your currently using any method of family planning?**	☐ **Yes**<br>☐ **No → Q2**
	**If yes, please mark the methods that you are current using:**	☐ **Pill** (Oral Contraceptives)<br>☐ **Condom**<br>☐ **Injectable** (Petogen/ Depo-Provera/ Nuristerate)<br>☐ **IUD** (Intrauterine Device)<br>☐ **Partner was sterilized/ vasectomy**
**Q2.**	**Are you currently trying to get pregnant?**	☐ **Yes**<br>☐ **No**
