# Supplementary figures and images for: Longitudinal patterns of unmet need for contraception among women living with HIV on antiretroviral therapy in South Africa
Source: PLoS One. 2018 Dec 20;13(12):e0209114. doi: 10.1371/journal.pone.0209114 (PMC6301780; doi:10.1371/journal.pone.0209114)

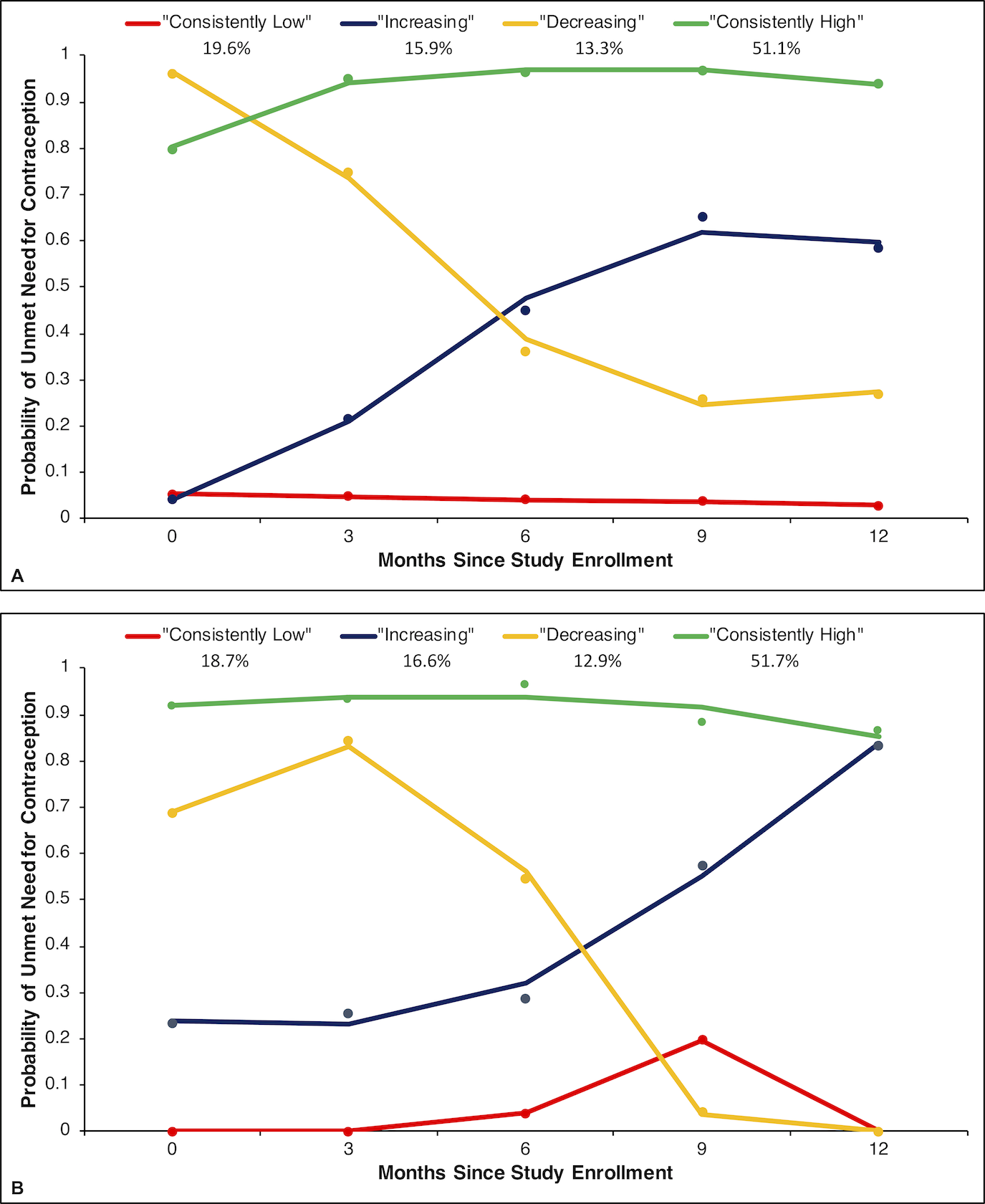

Supplement: S1 Fig — Figure that illustrates trajectories of unmet need for contraception estimated using quarterly assessments for A) the full cohort (N = 850) and B) recent ART initiators (N = 157). (TIFF) [file pone.0209114.s002.tiff]
