# Supplementary material for: Longitudinal patterns of unmet need for contraception among women living with HIV on antiretroviral therapy in South Africa
Source: PLoS One. 2018 Dec 20;13(12):e0209114. doi: 10.1371/journal.pone.0209114 (PMC6301780; doi:10.1371/journal.pone.0209114)
Supplement: S1 Table — (DOCX) [file pone.0209114.s003.docx]

**S1 Table. Characteristics of Women Assigned to “Consistently Low,” “Increasing,” “Decreasing,” and “Consistently High” Unmet Need Trajectory Groups using a Maximum-Probability Assignment Rule (N=850)**

|  | **"Consistently Low"** N=214 (25.2%) | | **"Increasing"** N=73 (8.6%) | | **"Decreasing"** N=116 (13.6%) | | **“Consistently High”**  N=447 (52.6%) | |
| --- | --- | --- | --- | --- | --- | --- | --- | --- |
|  | **Median** | **IQR** | **Median** | **IQR** | **Median** | **IQR** | **Median** | **IQR** |
| **Posterior probability of group membership** | 0.95 | 0.71-0.99 | 0.89 | 0.72-0.98 | 0.90 | 0.70-0.98 | 0.99 | 0.85-1.00 |
| **Age (years)**^*^ | 30.1 | 27-32 | 29.8 | 28-32 | 30.3 | 27-33 | 30.8 | 27-33 |
| **Income (ZAR)**^*^ | 2000.0 | 1000-3000 | 2500.0 | 1700-4200 | 2000.0 | 1005-3200 | 2000.0 | 1000-3500 |
| **No. living children**^*†^ | 1 | 0-2 | 1 | 0-2 | 1 | 0-1 | 1 | 1-2 |
| **CD4 count (cells/µl)**^*^ | 314.0 | 192-474 | 311.0 | 172-492 | 247.5 | 175-455 | 319.0 | 175-454 |
| **Months on ART**^*^ | 13.6 | 6-26 | 12.6 | 6-22 | 11.3 | 3-21 | 13.3 | 6-26 |
|  | **n** | **%** | **n** | **%** | **n** | **%** | **n** | **%** |
| **Lost to follow-up at 6 months** | 20 | 9.4 | 1 | 1.4 | 4 | 3.5 | 35 | 7.8 |
| **Became pregnant during follow-up** | 35 | 16.4 | 6 | 8.2 | 17 | 14.7 | 91 | 20.4 |
| **Unmet need at study enrollment** | 12 | 5.6 | 4 | 5.5 | 107 | 92.2 | 346 | 77.4 |
| **Planned to conceive next 12 months** | 109 | 50.9 | 40 | 54.8 | 76 | 65.5 | 148 | 33.1 |
| **In relationship with main partner ≥3 years**^*^ | 108 | 50.5 | 50 | 68.5 | 68 | 58.6 | 290 | 64.9 |
| **Has living children**^*^ | 158 | 73.8 | 52 | 71.2 | 77 | 66.4 | 379 | 84.8 |
| **Education completed**^*^ |  |  |  |  |  |  |  |  |
| None-Grade 10 | 58 | 27.1 | 17 | 23.3 | 33 | 28.5 | 110 | 24.6 |
| Grade 11-Grade 12 | 125 | 58.4 | 49 | 67.1 | 63 | 54.3 | 275 | 61.5 |
| Post-grad degree or certificate | 31 | 14.5 | 7 | 9.6 | 20 | 17.2 | 62 | 13.9 |
| **Social grant recipient**^*^ | 60 | 28.0 | 19 | 26.0 | 15 | 12.9 | 110 | 24.6 |
| **Employed**^*†^ | 115 | 53.7 | 44 | 60.3 | 79 | 68.1 | 272 | 60.9 |
| **Disclosed HIV status to main partner**^*†‡^ | 145 | 83.8 | 55 | 75.3 | 27 | 75.7 | 356 | 82.4 |
| **Any physical IPV with main partner**^*§^ | 18 | 10.5 | 7 | 9.6 | 9 | 8.2 | 39 | 9.1 |
| **Main partner desires a/another child**^*†^ | 145 | 67.8 | 63 | 86.3 | 104 | 89.7 | 332 | 74.3 |
| No | 28 | 16.2 | 10 | 13.7 | 7 | 6.3 | 100 | 23.2 |
| Yes | 131 | 75.7 | 59 | 80.8 | 92 | 82.3 | 287 | 66.4 |
| Unsure | 14 | 8.1 | 4 | 5.5 | 12 | 10.8 | 45 | 10.4 |
| **Problems with contraceptive method**^*†^ | 29 | 13.6 | 19 | 26.0 | 14 | 12.1 | 84 | 18.8 |
| **Provider discussed future childbearing**^*^ | 89 | 41.6 | 33 | 45.2 | 45 | 38.8 | 178 | 39.8 |
| **Provider discussed PMTCT**^*^ | 139 | 65.0 | 52 | 71.2 | 43 | 62.9 | 309 | 69.1 |
| **Provider discussed HC options**^*^ | 101 | 47.2 | 40 | 54.8 | 38 | 32.8 | 231 | 51.7 |
| **Provider discouraged having a/another child**^*^ | 9 | 4.2 | 7 | 9.6 | 5 | 4.3 | 32 | 7.2 |

Abbreviations; IQR: Interquartile Range, ART: Antiretroviral therapy, No.: Number, IPV: Intimate Partner Violence, HC: Hormonal Contraception, PMTCT: Prevention of Mother-to-child Transmission

^*^Assessed in stage 1 of predictor analysis

^†^Included in stage 2 of predictor analysis

^‡^n=789

^§^n=784
