# Supplementary material for: Longitudinal patterns of unmet need for contraception among women living with HIV on antiretroviral therapy in South Africa
Source: PLoS One. 2018 Dec 20;13(12):e0209114. doi: 10.1371/journal.pone.0209114 (PMC6301780; doi:10.1371/journal.pone.0209114)
Supplement: S2 Table — (DOCX) [file pone.0209114.s004.docx]

**S2 Table. Characteristics of Recent ART Initiators Assigned to “Consistently Low,” “Increasing,” “Decreasing,” and “Consistently High” Unmet Need Trajectory Groups using a Maximum-Probability Assignment Rule (N=157)**

|  | **"Consistently Low"** N=41 (26.1%) | | **"Increasing"** N=24 (15.3%) | | **"Decreasing"** N=21 (13.4%) | | **“Consistently High”**  N=71 (45.2%) | |
| --- | --- | --- | --- | --- | --- | --- | --- | --- |
|  | **Median** | **IQR** | **Median** | **IQR** | **Median** | **IQR** | **Median** | **IQR** |
| **Posterior probability of group membership** | 0.97 | 0.81-0.99 | 0.94 | 0.76-0.99 | 0.92 | 0.85-0.99 | 0.98 | 0.73-0.98 |
| **Age (years)** | 28.8 | 27-31 | 30.6 | 25-33 | 30.3 | 28-33 | 30.0 | 27-32 |
| **Income (ZAR)** | 1300.00 | 500-2000 | 2500.0 | 850-3500 | 1800.0 | 1000-4200 | 1800.0 | 1300-3000 |
| **No. living children** | 0 | 0-1 | 1 | 0-1 | 1 | 0-1 | 1 | 0-2 |
| **CD4 count (cells/µl)** | 132.0 | 71-192 | 165.0 | 110-179 | 151.0 | 105-186 | 140.0 | 68-175 |
| **Months on ART** | 1.9 | 1-2 | 0.7 | 0-2 | 0.9 | 0-2 | 1.0 | 0-2 |
|  | **n** | **%** | **n** | **%** | **n** | **%** | **n** | **%** |
| **Lost to follow-up at 6 months** | 4 | 9.8 | 1 | 4.2 | 1 | 4.8 | 7 | 9.9 |
| **Became pregnant during follow-up** | 9 | 22.0 | 2 | 8.3 | 0 | 0 | 14 | 19.7 |
| **Unmet need at study enrollment** | 1 | 2.4 | 11 | 45.8 | 18 | 85.7 | 64 | 90.1 |
| **Planned to conceive next 12 months** | 22 | 53.7 | 20 | 83.3 | 18 | 85.7 | 30 | 42.3 |
| **In relationship with main partner ≥3 years** | 8 | 19.5 | 14 | 58.3 | 13 | 61.9 | 44 | 62.0 |
| **Has living children** | 20 | 48.8 | 14 | 58.3 | 14 | 66.7 | 53 | 74.7 |
| **Education completed** |  |  |  |  |  |  |  |  |
| None-Grade 10 | 15 | 36.6 | 8 | 33.3 | 7 | 33.3 | 26 | 36.6 |
| Grade 11-Grade 12 | 24 | 58.5 | 11 | 45.8 | 12 | 57.1 | 38 | 53.5 |
| Post-grad degree or certificate | 2 | 4.9 | 5 | 20.8 | 2 | 9.5 | 7 | 9.9 |
| **Social grant recipient** | 6 | 14.6 | 4 | 16.7 | 1 | 4.8 | 12 | 16.9 |
| **Employed** | 17 | 41.5 | 12 | 50.0 | 12 | 57.1 | 42 | 59.2 |
| **Disclosed HIV status to main partner**^*^ | 21 | 84.0 | 16 | 69.6 | 18 | 85.7 | 57 | 87.7 |
| **Any physical IPV with main partner** | 2 | 8.3 | 0 | 0 | 3 | 14.3 | 10 | 15.4 |
| **Main partner desires a/another child**^†^ |  |  |  |  |  |  |  |  |
| No | 2 | 8.0 | 0 | 0 | 1 | 4.8 | 13 | 20.0 |
| Yes | 22 | 88.0 | 21 | 91.3 | 18 | 85.7 | 44 | 67.7 |
| Unsure | 1 | 4.0 | 2 | 8.7 | 2 | 9.5 | 8 | 12.3 |
| **Problems with contraceptive method** | 1 | 2.4 | 4 | 16.7 | 3 | 14.3 | 11 | 15.5 |
| **Provider discussed future childbearing** | 18 | 43.9 | 8 | 33.3 | 7 | 33.3 | 35 | 49.3 |
| **Provider discussed PMTCT** | 21 | 51.2 | 14 | 58.3 | 11 | 52.4 | 39 | 54.9 |
| **Provider discussed HC options** | 12 | 29.3 | 10 | 41.7 | 6 | 28.6 | 26 | 36.6 |
| **Provider discouraged having a/another child** | 5 | 12.2 | 1 | 4.2 | 1 | 4.8 | 1 | 1.4 |

Abbreviations; IQR: Interquartile Range, ART: Antiretroviral therapy, No.: Number, IPV: Intimate Partner Violence, HC: Hormonal Contraception, PMTCT: Prevention of Mother-to-child Transmission

^*^n=134

^†^n=145
